# Supplementary material for: HPV16 Load Is a Potential Biomarker to Predict Risk of High-Grade Cervical Lesions in High-Risk HPV-Infected Women: A Large Longitudinal French Hospital-Based Cohort Study
Source: Cancers (Basel). 2021 Aug 18;13(16):4149. doi: 10.3390/cancers13164149 (PMC8394477; doi:10.3390/cancers13164149)
Supplement: Supplementary file 1 [file cancers-13-04149-s001.zip › cancers-1280713-supplementary.pdf]

Supplementary Materials

HPV16 Load is a Potential Biomarker to Predict Risk of High-Grade Cervical Lesions in High-Risk HPV-Infected Women: A Large Longitudinal French Hospital-Based Cohort Study

Antoine Baumann, Julie Henriques, Zohair Selmani, Aurélia Meurisse, Quentin Lepiller, Dewi Vernerey, Séverine Valmary-Degano, Sophie Paget-Bailly, Didier Riethmuller, Rajeev Ramanah, Christiane Mougin and Jean-Luc Prétet

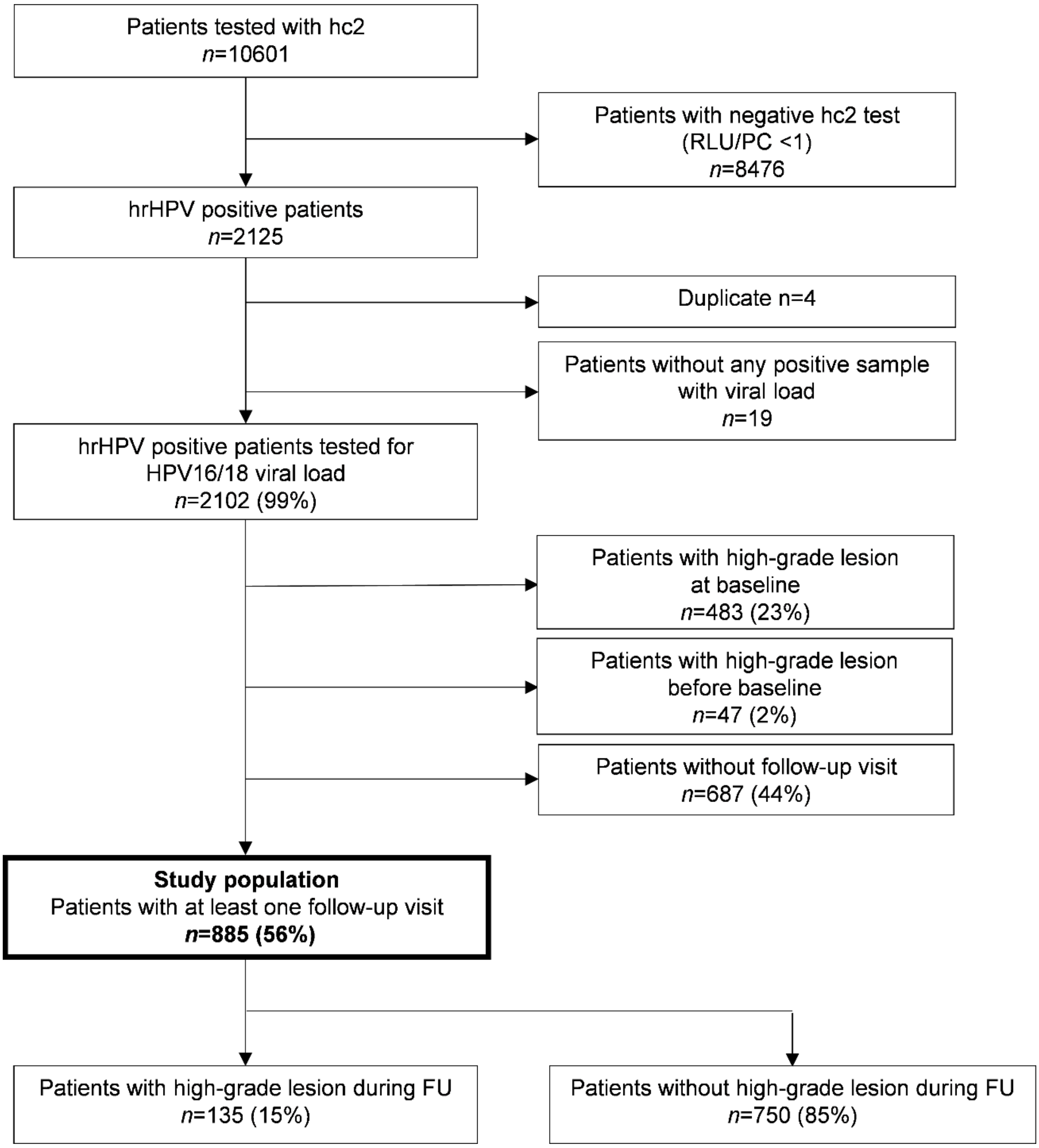

Figure S1. Flow chart for the hrHPV DNA test and HPV16/18 DNA load test performed on the study population. FU: Follow-Up; hc2: Hybrid Capture 2; hrHPV: High-Risk Human Papillomavirus; RLU/PC: Relative Light Unit/Positive Control.

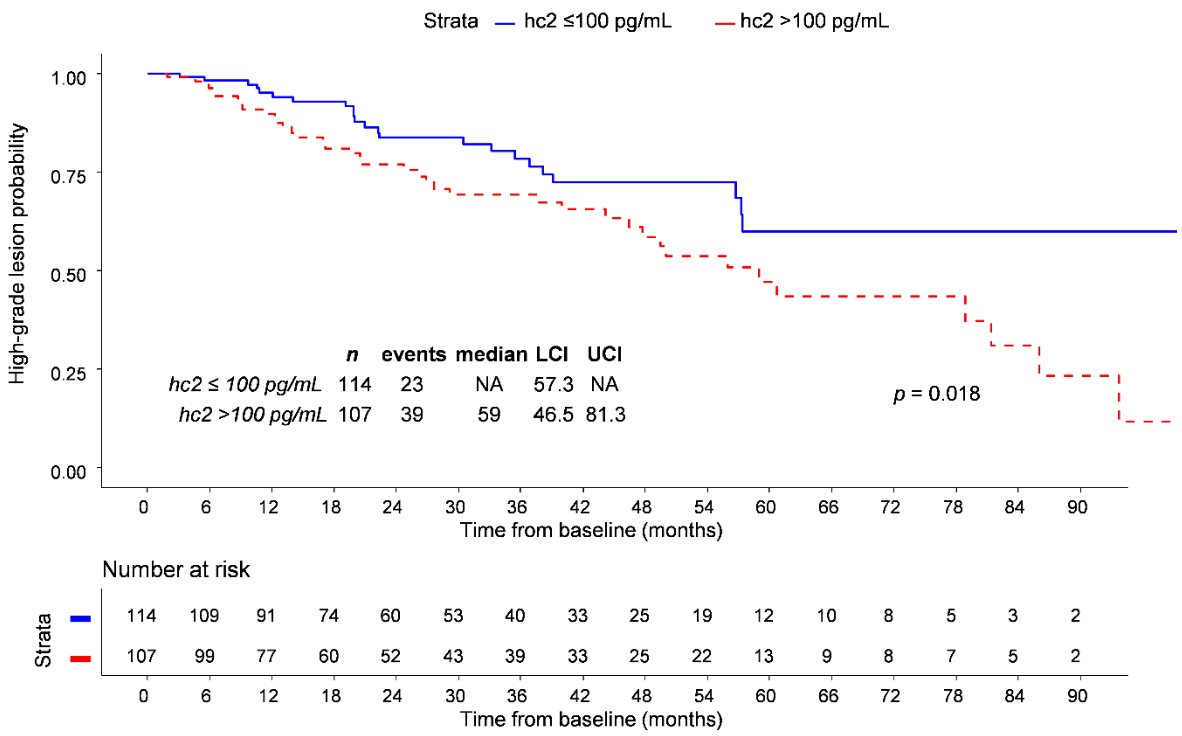

Figure S2. Kaplan–Meier estimates for time from baseline to the development of high-grade lesion according to hc2 results in HPV16 positive women. LCI: 95% Lower Confidence Interval; UCI: 95% Upper Confidence Interval. FU: Follow-Up; hc2: Hybrid Capture 2; hrHPV: High-Risk Human Papillomavirus; NA: Not Available.

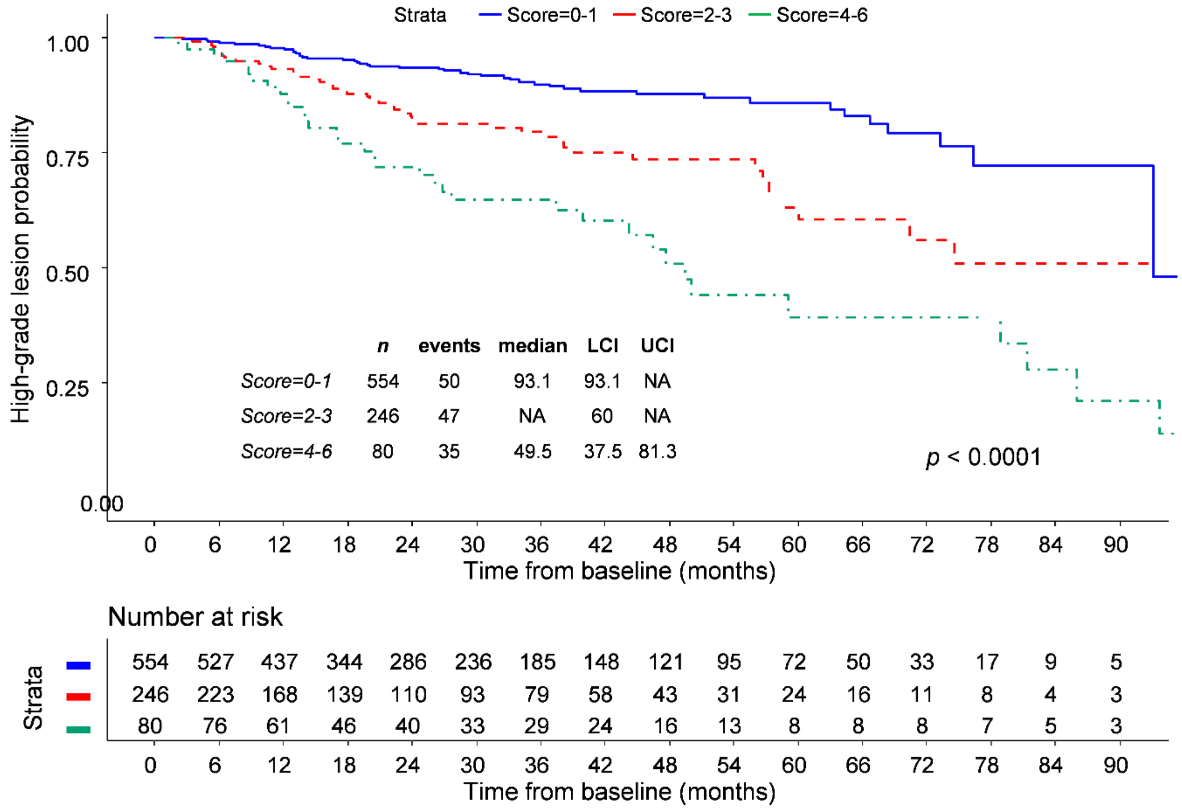

Figure S3. Kaplan–Meier estimates for time from baseline to the development of high-grade lesion according to prognostic score. LCI: 95% Lower Confidence Interval; UCI: 95% Upper Confidence Interval; NA: Not Available.

Table S1. Sequences of qPCR primer pairs and probes targeting a polymorphic region of HPV16 and HPV18 E6 genes and the cellular albumin gene.

Primer	Sequence
HPV16 Fw	5′-GAGAACTGCAATGTTTCAGGACC-3′
HPV16 Rv	5′-TGTATAGTTGTTTGCAGCTCTGTGC-3′
HPV16 probe	5′-FAM-CAGGAGCGACCCAGAAAGTTACCACAGTT-BHQ1-3′
HPV18 Fw	5′-ACACCACAATACCATGGCG-3′
HPV18 Rv	5′-TTCAGTTCCGTGCACAGATC-3′
HPV18 probe	5′-FAM-CAACACGGCGACCCTACAAGCTAC-BHQ1-3′
Albumin Fw	5′-GCTGTCATCTCTTGTGGGCTGT-3′
Albumin Rv	5′-AAACTCATGGGAGCTGCTGGTT-3′
Albumin probe	5′-FAM-CCTGTCATGCCCACACAAATCTCTCC-BHQ1-3′

Fw: Forward; HPV: Human Papillomavirus; qPCR: Quantitative Polymerase Chain Reaction; Rv Reverse

Table S2. Univariate cox model for the association between prognostic groups and high-grade lesion occurrence in the study population.

Score	*n* (Events)	HR	95% CI	*p*-Value
-	880 (132)	-	-	-
0–1	554 (50)	1	-	-
2–3	246 (47)	2.39	1.61–3.57	<0.0001
4–6	80 (35)	4.53	2.92–7.01	<0.0001

HR: hazard ratio; CI: confidence interval

© 2021 by the authors. Licensee MDPI, Basel, Switzerland. This article is an open access article distributed under the terms and conditions of the Creative Commons Attribution (CC BY) license (http://creativecommons.org/licenses/by/4.0/).
